# Relationship between *Leishmania* IFAT Titer and Clinicopathological Manifestations (Clinical Score) in Dogs

**DOI:** 10.1155/2014/412808

**Published:** 2014-06-03

**Authors:** Daniela Proverbio, Eva Spada, Giada Bagnagatti de Giorgi, Roberta Perego, Emanuela Valena

**Affiliations:** Dipartimento di Scienze Veterinarie per la Salute, la Produzione Animale e la Sicurezza Alimentare (VESPA), Università degli Studi di Milano, Via Celoria 10, 20133 Milano, Italy

## Abstract

During canine leishmaniasis (CanL) due to * Leishmania infantum,* high levels of antibodies production are associated with the presence of various clinical signs, because of the deposition of soluble immune complexes in organs and tissues. The immunofluorescence antibody test (IFAT) is one of the most commonly used techniques for detection of anti-*Leishmania* antibodies. The purpose of this study was to assess whether there is a correlation between clinical signs and IFAT titers in dogs naturally infected with * Leishmania*. A retrospective study was performed on medical records of 49 dogs diagnosed with CanL. Information extracted from the medical records of each dog with CanL was clinical score, IFAT titer, serum total protein (TP), gamma globulin (IgG) and creatinine concentration, and protein creatinine ratio in urine sample (UP/UC) at each follow-up examination. Results show that dogs with highest IFAT titers recorded had higher mean clinical scores indicating a positive relationship (*P* < 0.0001) between anti-*Leishmania* antibodies (IgG) and clinical manifestations, which becomes more evident in severe clinical forms of canine leishmaniasis. Higher TP and IgG serum concentrations were recorded in dogs with higher clinical scores. Significant association was observed between UP/UC and the IFAT titer (*P* = 0.004).

## 1. Introduction

Leishmaniasis due to* Leishmania *spp infection has a wide distribution in four continents and affects mainly dogs, humans, and rodents. Dogs are the main reservoir for zoonotic human visceral infection caused by* L. infantum* parasite and play a pivotal role in the transmission of the disease, via phlebotomine sandflies (*Phlebotomus* spp. and* Lutzomyia *spp. in the Old and New World), to people [1]. Canine leishmaniasis (CanL) due to* L. infantum* is a life-threatening disease, which may be fatal before treatment can be instigated [1]. Clinical presentations of CanL range from subclinical/asymptomatic to full-blown disease, with variable laboratory findings depending on the host's immune response [2]. When the immune response is mediated by Th2 lymphocytes, IL-4 secretors antibody production is high and this is associated with severe clinical manifestations [3]. Many studies [4, 5] report that high levels of antibodies production are associated with the presence of clinical signs such as epistaxis, proteinuria, polyuria, polydipsia, uveitis, and skin ulcers because of the deposition of soluble immune complexes in organs and tissues [3, 6]. Several authors [5, 7, 8] evaluated the profile of anti-*Leishmania* antibodies in dogs with different clinical forms of visceral leishmaniasis using methods not easily obtainable in clinical practice. The immunofluorescence antibody test (IFAT) is one of the most commonly used techniques [9] for detection of anti-*Leishmania* antibodies and is recommended by World Organization for Animal Health (OIE) as the reference serological method [10]. High antibody levels are associated with high levels of parasitism [2] and provide a definitive diagnosis of CanL [9].

The purpose of this study was to assess whether there is a correlation between clinical signs and IFAT titers in dogs, naturally infected with* L. infantum,* to determine whether IFAT titers could be used as a predictor of the severity of clinical disease.

## 2. Materials and Methods

A retrospective study was performed. The medical records of 131 dogs diagnosed with CanL that were presented to the Teaching Animal Hospital of the Department of Health, Animal Science and Food Safety of University of Milan between 2008 and 2013 were reviewed. The following inclusion criteria were used: clinical diagnosis of CanL confirmed by positive serology for Leishmania infantum using IFAT and cytological identification of* Leishmania* amastigotes or detection of parasite DNA using polymerase chain reaction (PCR). IgG anti-*L. infantum* antibodies were measured by IFAT according to the recommendations of OIE [10] using MHOM/IT/80/IPT1 as a whole-parasite antigen fixed on multispot slides (Bio Merieux Spa, Florence, Italy) and fluorescently labeled anticanine gamma globulin (Sigma Aldrich, Milan, Italy) as conjugate. Positive sera were serially diluted and tested to establish the maximum reaction titer, starting at a dilution of 1 : 40. Positive and negative controls were included on each slide.

The real-time PCR analysis of whole blood or samples from lymph node aspiration was performed using the Illustra Blood genomicPrep Mini Spin kit (GE Healthcare, Milan, Italy) following the manufacturer's instructions. The target for amplification was a 116-bp fragment in the constant region of the kDNA minicircle of* L. infantum*. This is one of the kDNA minicircle families that are used to identify the* Leishmania* genus. The primers used were QLK2-UP 5′-GGCGTTCTGCGAAAACCG-3′ and QLK2-DOWN 5′-AAAATGGCATTTTCGGGCC-3′; the TaqMan probes were Q Leish Probe 2 and 5′-FAM TGGGTGCAGAAATCCCGTTCA-3′-Black Hole.

At the moment of the diagnosis and at each follow-up a complete physical examination was performed on all dogs after which clinical assessment of the severity of signs attributable to* Leishmania* infection (scored on a scale from 0 to 3) was made (Table 1). During each follow-up a complete blood count, hematological and serum biochemical examinations (including the determination of total protein and serum electrophoretic pattern), and urine examination were performed.

All dogs in which concomitant infectious diseases (e.g., babesiosis, ehrlichiosis, and dirofilariasis) were diagnosed by parasitological or/and serological examinations were excluded.

### 2.1. Medical Records Review

Information extracted from the medical records of each dog at each follow-up examination with CanL was clinical score, IFAT titer, serum total protein (TP), gamma globulin (IgG) and creatinine concentration, and protein creatinine ratio in urine sample (UP/UC).

A total of 49/131 dogs (37.4%) met the criteria for inclusion in the study. Ages of the dogs ranged from 0.7 to 14 years with a median age of 6 years. Twenty-eight were intact males and 21 were females (10 neutered); 21 were X-breeds and 28 were purebreds. A mean of 13.9 (min 2-max 18) follow-up examinations was done for each dog.

To assess the relationship between the IFAT titer and the parameters considered, that is, clinical score, serum total protein, IgG and creatinine concentration, and UP/UC, the recorded values from each follow-up examination were divided into 9 groups according to the IFAT value (1 : 40, 1 : 80, 1 : 160, 1 : 320, 1 : 640, 1 : 1240, 1 : 2560, 1 : 5120, and 1 : 10240) regardless of the subject to which they belonged.

### 2.2. Statistical Analysis

Mean, standard deviation, median of clinical score value (CS), TP, IgG, creatinine (Cr), and UP/UC were calculated after calculating normal distribution of parametric data using the D'Agostino-Pearson test. Mann-Whitney was used for independent samples tests to assess any statistically significant difference in the mean of clinical scores and PT, IgG, creatinine, and UP/UC of dogs with different IFAT titers (40 versus 80, 80 versus 160, 160 versus 320, etc.).

Spearman's coefficient of rank correlation (rho) was used to evaluate the degree of association between the mean values of variables CS, PT, IgG, Cr, and UP/UC and IFAT titer.

For all tests significance was set as *P* < 0.05. Statistical analyses were performed using commercial software (MedCalc Software v.13.0.0.0, Mariakerke, Belgium).

## 3. Results

Comparison of mean values and standard deviation of clinical score, total protein, IgG, creatinine, and UP/UC in the 9 groups based on the IFAT titer are reported in Table 2.

The significance and the degree of association between the mean values of all variables, IFAT titer, clinical score, TP, IgG, creatinine, and UP/UC are reported in Table 3.

## 4. Discussion

Detection of specific serum antibodies is widely used in the diagnosis of CanL [11]. Treatment of sick dogs is often accompanied by a decrease in the specific antibody levels [9, 11, 12]. However, in other cases clinical improvement has not been associated with a decrease in the titer of specific antibodies [13].

This study was conducted to investigate whether there was a correlation between the IFAT titer and the clinical score in dogs with CanL.

Higher clinical score values were detected in dogs with higher IFAT titers and a significant level of association between IFAT titer and clinical score (*P* < 0.0001) was found. The dog with the maximum clinical score (score = 30) had the highest IFAT titer (1 : 10240).

There was a similar trend in serum total protein and IgG concentrations: higher TP and IgG concentrations were recorded in dogs with higher clinical scores. As expected, the IFAT titer was also related to TP and IgG (*P* < 0.0001). Similar results were found by Corona et al. [3] who reported that the changes in quantitative determination of specific antibodies paralleled those of total protein and IgG fractions.

It is well known that the clinical manifestations of CanL are the consequence of the host immune response and are associated with deposition of soluble immune complexes in different tissues [9]. In infected dogs, the defective cell-mediated immunity results in uncontrolled multiplication of the parasite and subsequent polyclonal, and sometimes monoclonal, activation of B cells with overproduction of immunoglobulins. These antibodies do not provide protection for the host but result in the formation of immune complexes that can damage a variety of tissues and organs. Several studies have described the levels of specific* Leishmania* IgG subclasses (IgG1, IgG2, IgM, IgA, and IgE) in sick, asymptomatic, and treated dogs, sometimes with conflicting results [4, 5, 7, 8]. Solano-Gallego et al. [14] determined the level of* Leishmania*-specific total IgG (IgG), IgG1, and IgG2 antibody responses in the sera of a wide range of canine populations: symptomatic and asymptomatic dogs from endemic areas and naturally and experimentally infected dogs, showing that clinical signs are directly related to IgG1 and IgG2 and that IgG2 concentrations are more strongly correlated with clinical illness than IgG1. De Freitas et al. [5] investigated the profile of anti-*Leishmania *antibodies in different clinical forms of CanL and observed that IgG2 and IgM were positively correlated with clinical signs while total IgG, IgG1, and IgA were negatively correlated. The varying immune response of infected animals may account for the conflicting results reported by various authors [4]. The IFAT test is one of the recommended methods for the evaluation of anti-*Leishmania *antibodies [15] and is widely used in clinical practice while the antibody assay of each class of antibodies is not routinely practiced. Our results are in accordance with the findings of Almeida et al. [6] and Vercammen et al. [16] who both reported that IgG anti-*Leishmania* titers increased significantly in symptomatic dogs when compared to an asymptomatic control group.

During CanL overproduction of IgG, IgM and IgA, consequent to B cell activation, causes the formation of immuno-complexes [17].

Immunomediated mechanisms have a pivotal role in the development of renal pathology [18], and immunocomplex deposition in glomeruli is the cause of four principal types of glomerulonephritis: focal or diffuse mesangial glomerulonephritis, membranous glomerulonephritis, membranoproliferative glomerulonephritis, and focal segmental glomerulosclerosis [19]. There is strong evidence that proteinuria is a risk factor for the development and progression of renal failure and that renal disease is the most severe complication of CanL and is a sign of poor prognosis [20]. For these reasons, we have correlated the value of creatinine and UP/UC with the IFAT titer to assess whether there was an association between renal function analyses and the anti-*Leishmania *antibodies (IgG).

Higher UP/UC mean value has been reported in dogs with IFAT titers of 1 : 1280 and significant association between UP/UC and IFAT (*P* = 0.004) but not between IFAT titer and creatinine was observed (Table 3). Our results are in accordance with Poli et al. [21] who, in a study of glomerular lesions in dog with CanL, found no correlation between renal involvement and anti-*Leishmania* titer and creatinine but found correlation between glomerular lesions and the amount of proteinuria. Glomerulopathy that characterized CanL causes proteinuria due to deposition of circulating antigen/antibody complexes; this fact may explain the correlation between UP/UC and IgG value found in our study [18]. Renal damage with less than 75 percent of nonfunctional nephrons may not cause increase of serum creatinine concentration [22]; therefore, elevation of this parameter during CanL may show up later than the presence of protein in the urine. This may justify the lack of correlation between IFAT titer and creatinine serum concentration found in our study.

## 5. Conclusions 

In conclusion, our results show that dogs with the highest IFAT titers recorded had higher mean clinical scores indicating a positive relationship (*P* < 0.0001) between anti-*Leishmania* antibodies (IgG) and clinical manifestations that becomes more evident in polisymptomatic subjects. Significant correlation was found between IFAT titers and urinary UP/UC.

The results of this study may help to clarify whether IFAT titers could be usefully included in the short-term followup of dogs with CanL. Results from the present study support the idea that high IFAT titers are related with the presence of multiple clinical symptoms suggesting a positive relationship between IFAT titer and clinical manifestations.

## Figures and Tables

**Table 1:** Score for clinical parameters (on a scale from 0 to 3, maximum total score 87) in dogs with CanL.

Clinical sign	0	1	2	3
Appetite	Normal	Slight decrease	Moderate decrease	Anorexia
Mentation	Normal	Slight depression	Depression	Prostration
Exercise intolerance	No	Slight	Moderate	Refusal to move
Weight loss	No	Slight	Moderate	Severe
Polyuria	No	Slight	Moderate	Severe
Polydipsia	No	Slight	Moderate	Severe
UP/UC	No	<1	>1 < 2	>2
Localized muscle atrophy (temporal muscles)	No	Slight	Moderate	Severe
Generalized muscle atrophy	No	Slight	Moderate	Severe
Lymphadenomegaly	No	1-2 nodes	>2 < 4 nodes	Generalized
Splenomegaly	No		Yes	
Conjunctivitis and/or blepharitis	No	Unilateral and slight	Bilateral or unilateral severe	Bilateral and severe
Uveitis and/or keratitis	No	Unilateral and slight	Bilateral or unilateral severe	Bilateral and severe
Pale mucous membranes	No	Slight	Moderate	Severe
Epistaxis	Never presented	Sporadic	Frequent	Persistent
Mouth ulcers or nodules	No	1 or 2 small ulcers or nodules	>2 small ulcers or nodules	>1/4 of oral cavity covered by ulcers or nodules
Vomiting	No	Sporadic	Frequent	Frequent with blood
Diarrhea	No	Sporadic	Frequent	Persistent
Lameness	No	Sporadic	Frequent	Constant
Erythema	No	<10% body surface or slight generalized erythema	10–25% body surface or moderate generalized erythema	>25% body surface
Dry exfoliative dermatitis	No	<10% body surface or slight generalized erythema	10–25% body surface or moderate generalized erythema	>25% body surface
Ulcerative dermatitis	No	1-2 ulcers	3–5 ulcers	>5 ulcers
Nodular dermatitis	No	1-2 nodules	3–5 nodules	>5 nodules
Sterile pustular dermatitis	No	1-2 pustules	3–5 pustules	>5 pustules
Alopecia	No	<10% body surface	10–25% body surface erythema	>25% body surface
Altered pigmentation	No	Localized	Multifocal	Generalized
Hyperkeratosis truffle and pads	No	Slight	Moderate	Severe
Generalized hyperkeratosis	No	Slight	Moderate	Severe
Onychogryphosis	No	Slight	Moderate	Severe

**Table 2:** Mean value and standard deviation of clinical score, total protein, IgG, creatinine, and UP/UC in the 9 groups of dogs categorized according to IFAT titer.

*N*° dogs	IFAT titer	Mean clinical score (±SD)	Mean total protein (±SD) g/dL	Mean IgG (±SD) %	Mean creatinine (±SD) mg/dL	Mean UP/UC (±SD)
19	1 : 40	0.94 ± 0.97	6.72 ± 0.69	10.71 ± 3.23	1.15 ± 0.35	0.29 ± 0.68
46	1 : 80	0.98 ± 1.2	6.82 ± 0.66	11.67 ± 3.5	1.1 ± 0.38	0.84 ± 1.48
54	1 : 160	1.48 ± 2.02	6.97 ± 0.7	13.44 ± 3.81	0.94 ± 0.4	0.58 ± 1.56
48	1 : 320	2.65 ± 2.89	7.27 ± 0.88	17.85 ± 7.4	0.94 ± 0.31	0.16 ± 0.27
37	1 : 640	4.32 ± 3.48	7.94 ± 1.28	25.79 ± 12.18	1.05 ± 0.7	0.85 ± 2
19	1 : 1280	5.10 ± 4.71	8.52 ± 1.43	31.69 ± 13.03	1.26 ± 1	1.34 ± 2.27
9	1 : 2560	4.78 ± 3	9.38 ± 1.39	44.37 ± 9.41	1.62 ± 1.42	0.17 ± 0.06
2	1 : 5120	16 ± 9.9	10.75 ± 0.07	64.65 ± 17	0.95 ± 0.35	—
1	1 : 10240	30	10.7	53.2	0.7	—

**Table 3:** *P* and rho values of IFAT titer and clinical score, total protein, gamma globulin, creatinine, and UP/UC. *P* value shows the level of significance of the association and rho the degree of association between the mean values of variables.

	*P*	Rho
**IFAT**	<0.0001	0.454
Clinical score		

**IFAT**	<0.0001	0.532
Total protein		

**IFAT**	<0.0001	0.728
Gamma globulin		

**IFAT**	0.0786	−0.117
Creatinine		

**IFAT**	0.004	0.222
UP/UC		

**Total Protein**	<0.0001	0.436
Clinical score		

**Total protein**	<0.0001	0.678
Gamma globulin		

**Total protein**	0.0037	−0.193
Creatinine		

**Total Protein**	0.0307	0.168
UP/UC		

**Gammaglobulin**	<0.0001	0.391
Clinical score		

**Gammaglobulin**	0.95	0.0042
Creatinine		

**Gammaglobulin**	0.0243	0.175
UP/UC		

**Creatinine**	0.0148	−0.162
Clinical score		

**Creatinine **	0.8171	0.0181
UP/UC		

**UP/UC**	0.0035	0.225
Clinical score		
